# Soft Tissue Management in Implant Dentistry: A Comprehensive Review

**DOI:** 10.7759/cureus.79557

**Published:** 2025-02-24

**Authors:** Sumeet Agarwal, Sanpreet S Sachdev, Laresh N Mistry, Sneh Agrawal, Shantanu Deshpande, Vivek Sharma, Rohit Thorat

**Affiliations:** 1 Prosthodontics, Bharati Vidyapeeth (Deemed to be University) Dental College and Hospital, Navi Mumbai, IND; 2 Oral Pathology and Microbiology, Bharati Vidyapeeth (Deemed to be University) Dental College and Hospital, Navi Mumbai, IND; 3 Pediatric and Preventive Dentistry, Bharati Vidyapeeth (Deemed to be University) Dental College and Hospital, Navi Mumbai, IND; 4 Periodontology, Bharati Vidyapeeth (Deemed to be University) Dental College and Hospital, Navi Mumbai, IND; 5 Prosthodontics, Bharati Vidyapeeth (Deemed to be University) Dental College and Hospital, Pune, IND

**Keywords:** esthetic implant outcomes, implant dentistry, keratinized tissue, mucogingival management, osseointegration, peri-implant mucosa, soft tissue augmentation

## Abstract

The success of dental implant therapy relies on achieving optimal osseointegration while maintaining the esthetics and function of the surrounding soft tissues. Soft tissue management plays a crucial role in ensuring long-term implant stability, preventing complications, and enhancing esthetic outcomes. The peri-implant mucosa differs structurally and functionally from the gingiva surrounding natural teeth, necessitating specific augmentation strategies to compensate for tissue deficiencies. Factors such as biotype, mucogingival conditions, wound healing dynamics, ridge deficiencies, general patient health, genetics, and oral habits such as smoking influence treatment outcomes. Various soft tissue augmentation techniques, including pedicle grafts, free connective tissue grafts, pouch grafts, and onlay grafts, have been employed to enhance peri-implant soft tissue volume and contour. These procedures improve keratinized mucosa width, reduce mid-buccal recession, and contribute to superior pink esthetic scores. While immediate and delayed soft tissue augmentation protocols exist, the optimal timing remains debated. Clinical evidence suggests that augmenting soft tissue in conjunction with implant placement improves long-term esthetic outcomes and minimizes peri-implant bone loss. The present review highlights the significance of soft tissue augmentation in implant dentistry, emphasizing its role in improving peri-implant health, function, and patient satisfaction in esthetically demanding cases.

## Introduction and background

The primary objectives of any treatment procedure in dentistry include the restoration of the form, function, and esthetics of the oral structures, which comprise teeth, bones, and soft tissue. The field of implant dentistry is no stranger to these requirements. The success of dental implant treatment is largely dependent on osseointegration [1]. While restoring the function is crucial, the need for "perfect" esthetics cannot be overlooked, even for dental implants.

An ideal result is when the clinical appearance of the tissues in the area restored by implants closely mimics the normal mucogingival contours. A successful implant therapy would optimally re-create the lost mucogingival contours while maintaining their relationship to the prosthesis by replacing the missing teeth [2]. Ideal conditions for implant placement are almost always not present, and therefore, there arises a need for the management of soft tissue at various stages of placement of implant so that long-term success is achieved. 

The crucial role of "thick versus thin" gingiva when treatment is restorative in nature was explained by Ochsenbein and Ross [3]. Periodontal biotypes, which were thicker in nature (85%), were found to be more predominant than thin scalloped biotypes (15%), as reported by a population-based study [4]. Kao et al. highlighted the significance of different biotypes of the gingiva in the planning of implant therapy [5]. These biotypes depict different architectures with regard to their gingival and osseous components. Moreover, they show varied pathological responses when any inflammatory, traumatic, or surgical insult is implicated in them. Their response to extraction trauma is also variable in pattern osseous remodeling. Appropriate precautionary measures ensure minimal resorption and a favorable environment for implant procedures, especially in thin alveolar plate cases, which are at high risk of remodeling [3,5].

Management of soft tissue based on biological concepts in implant-oriented dentistry was explored by Yeung in 2008. It was concluded that a good esthetic finish, adequate blood supply, and hard tissue support are of utmost importance. Preservation and recreation of lost alveolar bone play significant roles. Furthermore, the availability of a good keratinized gingival band around the implant best suits the implant abutment [6]. Dolanmaz et al. suggested the use of autogenous bone grafts, usually harvested from the mandibular symphysis of the ramus area [7].

In 2018, Hämmerle and Tarnow studied the etiology of deficiencies in soft tissue that are encountered during implant treatment, and these are systemic diseases, conditions, tissue healing, turnover rate, response, trauma, local factors, morphology and phenotype, biomechanical factors, and iatrogenic factors [8]. They may occur alone or as a combination. The more the factors, the greater the severity of soft tissue deficiency. These should be identified and treated appropriately.

Thoma et al. summarized that the conjunction of soft tissue augmentation with immediate as well as delayed implant placement ultimately leads to superior esthetics [9]. It has a limited effect on marginal bone levels. Parameters relevant clinically, namely, recession of the mucosa and gingival index along with plaque control, are better when the width of keratinized mucosa increases. Pink Esthetic Score and Complex Esthetic Index are widely used esthetic indices for implants which are single in number. Patient-reported outcome measures (PROMs) have limited response. Gharpure et al. provided an evidence-based, structured algorithm that places its emphasis on prior evaluation of keratinized tissue for implant placement procedures [10].

The following review presents a comprehensive description of various aspects involved in the management of soft tissue in implant dentistry.

## Review

Methodology

A comprehensive literature search was performed using the keywords "soft tissue management", "soft tissue augmentation", "periodontium", "flap", "ridge resorption", and "dental implants" in the databases PubMed, Embase, Web of Science, and Google Scholar to identify articles concerning soft tissue augmentation in the field of implant dentistry. Relevant articles with full text available in the English language published from inception up to January 2025 were identified. The literature was analyzed and compiled by all the authors, and a narrative synthesis was performed.

Oral mucous membrane (OMM): in health

OMM is defined as the moist lining in the oral cavity that is in continuation with the exterior surface of the skin on one end and the esophagus on the other. It comprises the buccolabial mucosa, gingiva, floor of the mouth, tongue, as well as hard palate [11]. Different parts of OMM have different turnover rates, which are directly related to wound healing and replacement of the lost tissues (Table 1). These also have implications post-extraction and in implant therapy.

**Table 1:** Turnover rates of different parts of the oral mucous membrane in comparison to skin

Region	Median (in days)	Range (in days)
Skin	27	22-75
Buccal/labial mucosa	14	5-16
Floor of the mouth	20	12-24
Hard palate	24	15-30
Attached gingiva	10	8-40
Junctional epithelium	6	4-10

A thorough knowledge of the normal anatomy of oral structures is very crucial in implant therapy. The gingiva is coral pink and scalloped; in contrast, the alveolar mucosa is red, smooth, and shiny in appearance [3-5]. The difference between the two gingival biotypes is given in Table 2.

**Table 2:** Characteristic differences between the gingival biotypes Width of 1.6-1.9 mm is not accounted for.

Thick gingival biotype	Thin gingival biotype
Width >2 mm	Width <1.5 mm
Bony architecture and soft tissue that are comparatively flat	Bony architecture and soft tissue that are highly scalloped
A wide band of keratinized tissue with short papillae	A narrow, keratinized gingival band that could terminate in a mucogingival junction which is wavy in nature
Soft tissue is dense and fibrotic	Soft tissue is delicate and friable
Comparatively big amount of gingiva connected	Minimal amount of attached gingiva
Thick osseous structure underneath	Bony dehiscence and fenestration are characteristics of thin underlying bone
Able to withstand acute trauma	Less resistant to trauma
Responds to illness by developing infrabony defects and pockets	Responds with gingival recession to insults and diseases
A distinct bundle of bone surrounding the tooth may be able to be accommodated by the thickness of the buccal plate	The buccal plate and bundle bone being one and the same bit of bone

**Figure 1:**
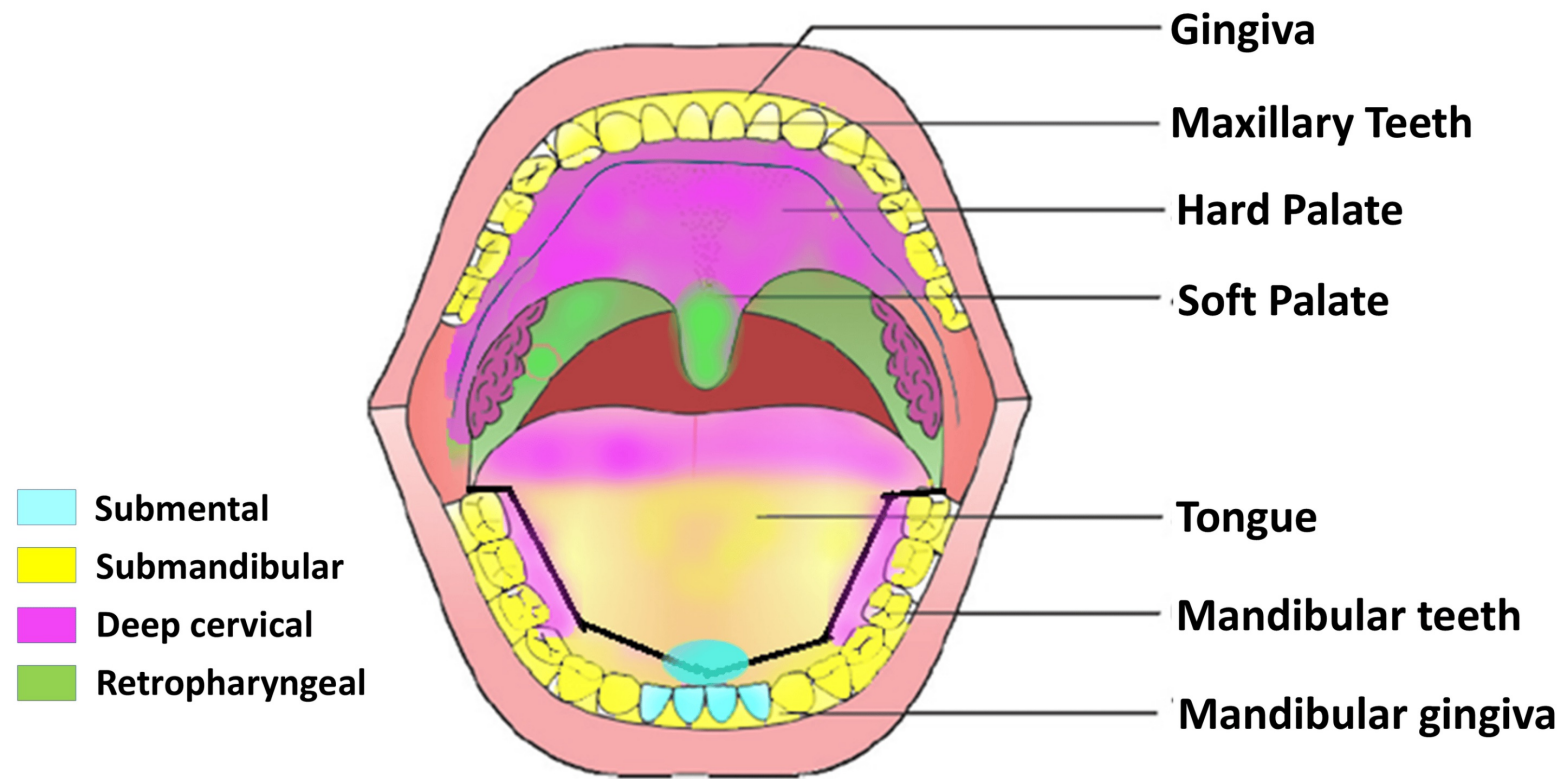
Lymphatic drainage of the different areas of the oral cavity into different groups of lymph nodes Maxilla: buccal gingiva and teeth, submandibular; palatal gingiva and hard and soft palate, deep cervical; soft palate, retropharyngeal Mandible: incisors, submental; canines to molars, submandibular; buccal gingiva, submandibular; lingual anterior teeth, submandibular; posterior teeth, deep cervical Tongue: tip, submental; anterior two-thirds, submandibular; posterior one-third, deep cervical Tonsils: lingual and palatal, deep cervical; tubal and pharyngeal, retropharyngeal Image Credits: Dr. Sanpreet S. Sachdev

Blood supply to the gingiva is through the supraperiosteal arterioles, periodontal ligament (PDL) vessels, and arterioles emerging from the crest of interdental septa. Lymphatic drainage of the different areas of the oral cavity is depicted in Figure 1 [12].

The integrity of the periodontium is maintained when a tooth is present in its socket, and the proprioception is provided by PDL and alveolar bone. Tooth loss causes hindrance in mechanical stimulation and consequent loss of trabeculation due to osteoclastic resorption [13]. Naturally, the scalloped contours and architecture of the overlying gingival tissues are also lost simultaneously, resulting in a reduced gingival profile. The response also varies according to the gingival biotype. The respective soft tissue and bone responses of both biotypes to inflammation, injury, or extraction are tabularized in Table 3.

**Table 3:** Response of the gingival biotypes to inflammation, surgery, and extraction

Event	Thick gingival biotype	Thin gingival biotype
Inflammation	An inflammatory radius of 2 mm will harm the cementum ligament and bundle bone because the alveolar housing surrounding the tooth is thicker	Involves every structure and causes resorption quickly
	Soft tissue: edematous/fibrotic alterations, bleeding upon probing, cyanosis, and marginal inflammation	Soft tissue: gingival recession and thin marginal redness
	Hard tissue: infrabony flaws and pocket development together with bone loss	Hard tissue: soft tissue recession linked to rapid bone loss
Surgery	Predicable soft and hard tissue contour after healing. More rebound of margins after crown-lengthening procedures	Where the tissue will recover and stabilize is difficult to predict. Further recession following crown-lengthening operations
Extraction	Minimal ridge atrophy, less alveolar deficiency, more predictable, and less demanding	Collapse of socket and ridge resorption in the apical and lingual direction resulting in contour deficiency+need for bone grafting

**Figure 2:**
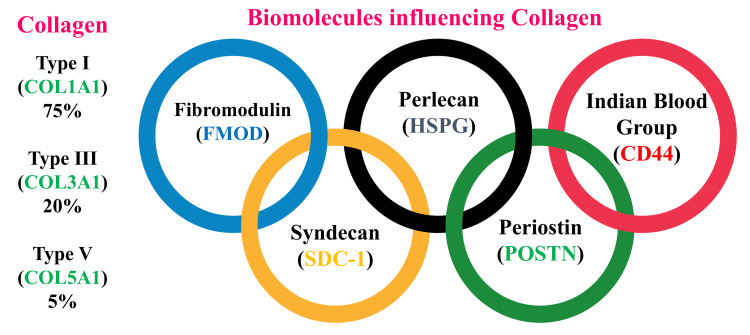
Attachment biomolecules in the periodontium influencing the adhesion, proliferation, and cross-linking of collagen fibers The figure denotes the types of collagen present in the periodontal tissues and the biomolecules influencing the adhesion, proliferation, and cross-linking of collagen fibers. Image Credits: Dr. Sanpreet S. Sachdev

The secondary wound healing process takes over to heal the socket. The main aim of repair is the formation and replenishment of bony tissue to initiate the osseous matrix formation [14]. The osteoid is subsequently calcified and undergoes maturation for a period of six weeks. The first test of the maturation is noticed at the base and on the outskirts of the socket. By the end of about four months, the newly formed bone completely fills the socket covered by an intact stroma and epithelium, and the osteogenic activity stops [15]. The different genes that maintain the balance between reabsorption and bone formation are mentioned in Figure 2. These genes are influenced by different proteins, for formation and reabsorption (Table 4) [16]. 

**Table 4:** Factors derived from the periodontal ligament regulating the formative and resorptive processes in bone remodeling

Bone formation	Bone resorption
Bone morphogenic proteins (BMPs)	Interleukins (IL-1, IL-4, IL-6)
Platelet-derived growth factor (PDGF)	Tumor necrosis factors (TNF-α, TNF-ß)
Epidermal growth factor (EGF)	Bacterial products (lipoteichoic acid)
Fibroblast growth factors (FGF-4, FGF-8)	Matrix metalloproteinases (MMPs)
Osteoprotegerin	Osteocalcin
Osteonectin	Osteopontin
Bone sialophosphoproteins	Hyaluronic acid
Chondroitin sulfates	Heparan sulfates

**Figure 3:**
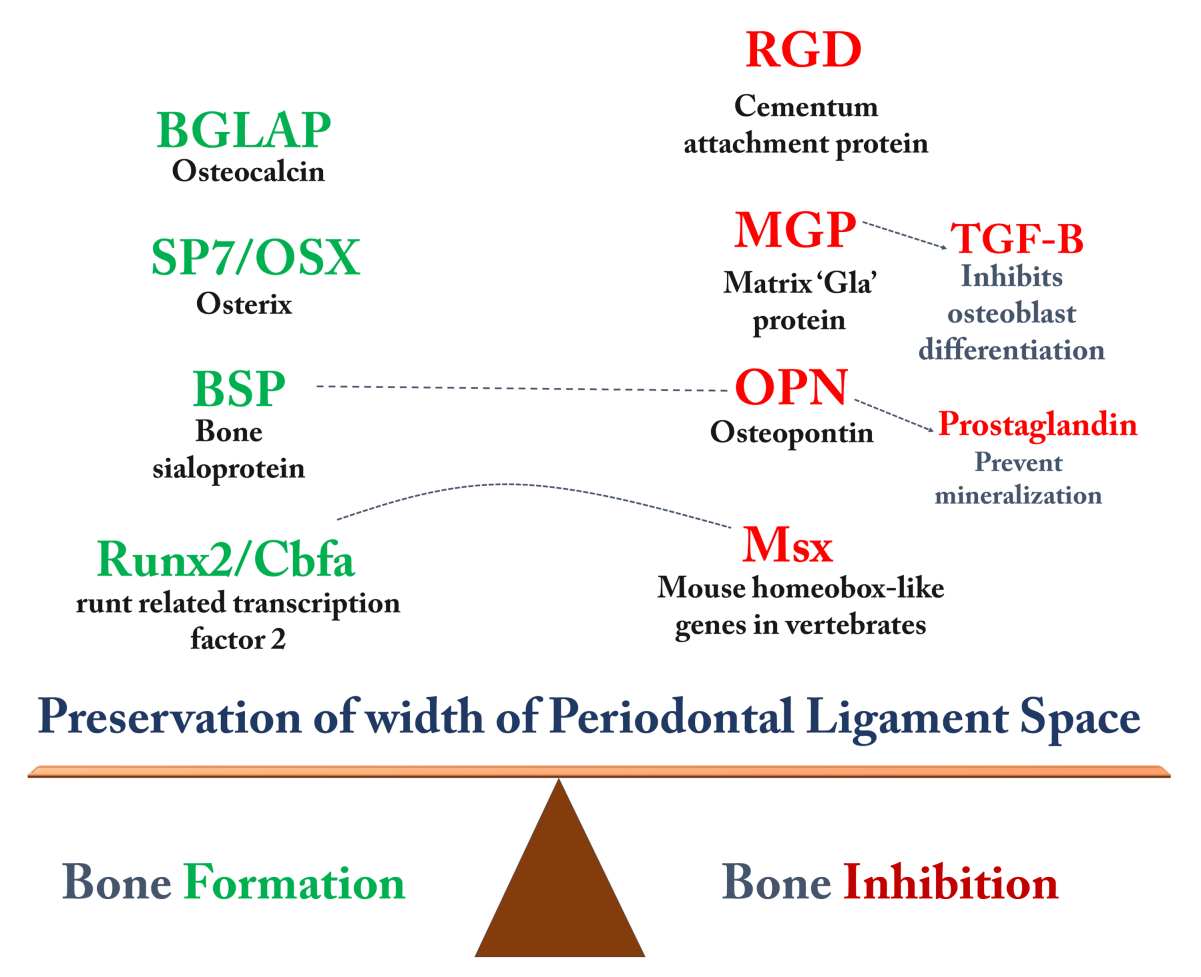
Genetic factors affecting bone remodeling in the periodontium The figure denotes the factors balancing the width of the periodontal ligament space. Red-colored biomolecules cause bone resorption, while green-colored biomolecules cause bone formation. Image Credits: Dr. Sanpreet S. Sachdev

Besides these, systemic diseases such as diabetes, medications such as bisphosphonates, smoking, and genetic factors are also responsible for increasing the severity and rate of bone loss [17]. The factors that maintain the attachment of the PDL to the bone and hence the thickness of the PDL are depicted in Figure 3. Fibromodulin is a proteoglycan that regulates collagen assembly, and syndecans and CD44 aid adhesion and proliferation. Perlecan plays an important role in controlling the bioavailability of growth factors, and periostin helps stabilize collagen cross-linking, which is important for wound healing [18]. 

Time of implant placement: a crucial decision

The time interval between the extraction of a tooth and the placement of an implant plays a vital role in esthetic as well as functional success. Due to severe resorption in the first year of tooth loss, implant placement should be done in this period. It can be of three varieties: immediate, at the time of extraction; early, within two months after extraction; and delayed, six months following the extraction [19]. Certain factors may be considered while deciding the time for implant placement (Table 5).

**Table 5:** Factors associated with timing of implant insertion

Time of implant insertion			
Factors	Immediate	Early	Delayed
Placement	At the time of extraction	Within 2 months of extraction	6 months following the extraction
Primary advantage	Reduces the number of surgical procedures	Allows the pathologies to subside	Allows adequate healing
Limitations	Cannot be placed in sockets with inadequate bone or existing pathologies	Healing may not be always achieved	Significant loss of hard and soft tissues
Treatment time	Drastically reduced	Short	Longer
Adjunctive antibiotics	Recommended	Optional	Seldom required
Implant positioning	Decisive	Dictated by socket to some extent	-
Resorption	Least	Less	Drastic
Need for bone substitutes	More frequent	Moderate	Less frequent
Requirement for stabilization	Apical and lateral	Apical/lateral	Optional

Difference between tissues surrounding implant and teeth

Brånemark defined osseointegration as "a direct structural and functional connection between ordered living bone and the surface of the load-covering implant" [20]. The principle of osseointegration underlies the basis of implant therapy. To achieve optimal osseointegration, it is imperative to have primary stability with minimal micromovement that may disrupt the integrative process. The concept is very similar to that of a healing fracture. Excess or uncontrolled mechanical loads could contribute to marginal bone loss, leading to failure of bone modeling and, ultimately, of the implant [21]. Fibrous tissue may also be formed in the interface between the implant and the bone. As a result, conventionally, a certain healing period was deemed necessary prior to the functional loading of the placed implants to facilitate healing and minimize these micromovements while attaining maximal osseointegration [22]. When considering post-placement healing and osseointegration, it is imperative to understand the difference between the environment present around the dental tissues and an implant [23]. The anatomy of the peri-implant mucosa is not exactly the same as the gingiva around natural dentition (Table 6).

**Table 6:** Difference between the environment of root and implant surface PDL: periodontal ligament

	Root surface	Implant surface
Collagen fibers	Perpendicular disposition	Parallel to implant surface
Attachment of fibers	To radicular cementum	Absent
PDL	Present	Absent
Vascular supply	Rich; from the plexuses present in the PDL	Diminished; only from the supraperiosteal vessels
Susceptibility to infections	Absent or low	High
Maintenance needs	Routine	Higher

Alveolar ridge and soft tissue defects

Identification and correction of mucogingival defects are critical for achieving optimal esthetic results in implant treatment. Should a prosthodontic treatment be attempted in a site with compromised quality of alveolar bone and surrounding tissues, several associated problems would occur. The three-dimensional defect causes the gingiva's scalloped, arch-shaped course to disappear while the papillae stay in place. As a result, "black" interdental gaps emerge. These lead to more food impaction in addition to esthetic and phonetic problems [24]. Alveolar ridge resorption after tooth extraction or implant loss results in a reduction of the buccal prominence of the gingiva and the alveolar mucosa. If the ridge defect is not surgically addressed, it causes reconstructive issues in the pontic area of a fixed restoration. In extreme circumstances, a detachable restoration would be required to correct the issue. The alveolar mucosa and gingiva may be scarred. The alveolar mucosa becomes smooth and glossy if the gingiva is overly thin [25].

Classification of localized alveolar ridge defects

Localized alveolar ridge defects can be classified qualitatively, based on their form in three dimensions, and semi-quantitatively, based on their severity and extent [26,27]. The magnitude of a ridge defect has been used to semi-quantitatively assess its severity. Less than 4 mm is considered a mild tissue defect, 3-6 mm is considered moderate, and more than 6 mm is considered substantial [28]. The ridge defect's dimensions are not described by this categorization. A more thorough semi-quantitative classification that assesses the tissue defect independently in both its vertical and horizontal dimensions is thus suggested [29].

From the deepest point of the tissue defect to an imaginary tangent that passes through the ends of the neighboring teeth's papillae, the vertical component of the defect is measured. The distance between the deepest point of the defect and an imaginary dental arch line that crosses the buccal cementoenamel junction or the mucogingival edge of two neighboring teeth is known as the horizontal extent. The semi-quantitative definition of the esthetically ideal ridge form is determined by the contour of the dental arch line and the imaginary tangent over the ridge defect, which in turn determines the augmentation volume. Ridge defects can also be categorized as one-, two-, three-, or four-tooth defects, according to how many teeth need to be replaced prosthetically.

Correction of the localized ridge defects

The deformity may be left uncorrected surgically and compensated for with a fixed prosthesis, or it may be rectified surgically during the pre-prosthetic preparation phase [30]. The procedures can be classified based on the method utilized for ridge augmentation into those requiring tissue augmentation and those requiring hard tissue augmentation.

Soft tissue augmentation

Patients who are partially or completely edentulous frequently receive soft tissue augmentation procedures prior to, during, and following implant implantation [31,32]. According to another recent study, these treatments could eliminate or reduce the need for hard tissue augmentation surrounding dental implants [33]. Basic flap design principles include avoiding anatomical structures, making the base wider than the apex, ensuring adequate width for maximum visibility, handling the flap delicately without tension, and starting the vertical releasing incision at the buccal vestibule and ending at the interdental papilla rather than the buccal or labial surface.

Approaches for soft tissue augmentation

The various approaches to the utilization of soft tissues for ridge augmentation can be classified as follows: (1) pedicle graft procedure, such as roll flap procedure, and (2) free graft procedures, such as pouch graft procedure, interpositional graft procedure, and onlay graft procedure. Studer et al. [29] advocated the use of the pedicle graft operation for the restoration of a single-tooth ridge defect with little horizontal and vertical loss; however, in cases of bigger defects, submerged free connective tissue graft procedures should be utilized.

Roll Flap Procedure

It involves a subepithelial pouch in which a preparation of de-epithelialized connective tissue pedicle graft is placed. It is effective in treating mild to moderate Class I ridge defects, especially in cases of single-tooth spaces. It enables the augmentation of tissue apically as well as labially to the pontic cervical area of the pontic, giving the recipient site a normal gingival-tooth interface. This gives a ridge convexity resembling the eminence produced by the roots of the adjacent tooth of a previously buccolingual ridge concavity [34].

From the palatal side, a pedicle of connective tissue in a rectangular shape is prepared. The amount of apicocoronal augmentation that is planned should match the length of the pedicle. First, the donor site's palatal surface epithelium is removed. A maximal amount of supraperiosteal connective tissue is raised from the palate utilizing aggressive dissection. The void created at the donor location will progressively fill up with granulation tissue.

When dissecting the pedicle flap, proceed with caution to avoid tissue perforation as the plane of dissection approaches the labial surface of the ridge. To preserve as much connective tissue and blood supply as possible at the recipient site, the dissection should be performed as close to the facial bone's periosteum as possible. As part of the trial procedure, the pedicle is tucked into the pouch. The pedicle size should now be adjusted. Once fitted, the pedicle is ready for stabilizing suture. The suture must be positioned close to the mucobuccal fold. This allows the surgeon to pull the pedicle to the apical part of the pouch. The suture should not be tightly tied because it only acts as a positioning and stabilizing device. The use of a resorbable suture material is recommended.

Pouch Graft Procedure

To produce the appropriate ridge contour, a free graft of connective tissue is inserted into a subepithelial pouch that has been formed in the area of the ridge deformity. There are several methods for creating the entrance incision and the dissection plane [35]. Coronal-apically, a horizontal incision is made on the palatal or lingual side of the defect, and the plane of dissection is carried in an apical direction. Apical-coronally, a horizontal incision is made high in the vestibule near the mucobuccal fold, and the dissection plane is followed coronally to the ridge crest. Later, one or two vertical entry cuts begin on both sides of the defect. The dissection plane is made laterally over the deformity radius.

Class I defects are fixed using this technique. Thin palatal tissues in patients with large-scale abnormalities may not be enough to supply the volume of donor tissue required for the deformity. In such cases, it is possible to select a variety of methods for strengthening the hard tissue. Starting well to the palatal/lingual side of the defect, the mesiodistal entrance incision for the pouch's edge should have a lengthy bevel. The facial tissue is stretched once the graft is filled in the punch.

The palatal edge of the flap can slide toward the facial surface without creating a gap at the incision line due to the entrance incision's long bevel. It is occasionally necessary to make vertical releasing incisions lateral to the border of the defect. Using a "trap-door" technique, a connective tissue-free graft is removed from a suitable donor site in the palate, tuberosity area, or edentulous area. The graft is promptly moved and positioned correctly at the recipient location. Sutures are used to seal the releasing and palatal entry incisions.

Interpositional Graft Procedure

Unlike subepithelial connective tissue grafts, interpositional grafts are not entirely covered and buried. Consequently, the epithelium on the donor tissue's surface does not need to be removed. A section of the graft needs to be placed above the surface of the tissue surrounding the recipient site if augmentation is needed in both the buccolingual and apicocoronal directions. As a result, some of the transplanted connective tissue will show through in the mouth [36]. Class I faults as well as minor and moderate Class II defects are corrected using interpositional graft treatments.

At the facial surface of the defect area, an envelope flap, also known as a split-thickness flap, is produced with releasing incisions. When estimating the amount of tissue that will need to be grafted to complete the deficiency, the temporary bridge is positioned to act as a guide. The length, width, and depth of the pouch's void can all be measured with a periodontal probe. A free graft of epithelium-connective tissue is removed from an appropriate donor site in the tuberosity or palate.

After being moved to the recipient site, the donor tissue is positioned there. The epithelial surface of the graft is positioned flush with the surrounding epithelium if the increase in ridge height is not desired. The graft is sutured to the recipient site's tissues all the way around. The pontics are trimmed and adjusted, and the temporary bridge is positioned.

A specific area of the graft must remain above the surface of the surrounding tissue if an increase in ridge height is also desired. The granulation tissue that forms during the healing process will eventually smooth and correctly epithelialize the boundary between the neighboring tissue and the graft. The ridge's profile will be shaped in part by the postoperative swelling.

Onlay Graft Procedure

The purpose of the onlay operation was to increase ridge height by augmenting ridge flaws in the apicocoronal plane. After being placed, onlay grafts, which are epithelialized free grafts, are nourished by the recipient site's de-epithelialized connective tissue. The initial thickness of the graft, the course of the wound healing process, and the amount of graft tissue that survives all affect how much apicocoronal augmentation is possible. To progressively raise the ridge height, the process can be repeated every two months if needed [37]. Large Class II and III lesions are treated with onlay graft operations. They are not appropriate in regions where scar tissue formation from prior wound healing has limited the recipient site's blood supply.

At the recipient location, every effort must be made to preserve as much lamina propria as feasible. To minimize vasoconstriction at the recipient site, the anesthetic fluid should be positioned high in the palate and vestibular fornix. The epithelium is cut off using a scalpel blade. Short, saw-like strokes are used to slide the scalpel across the recipient site at a level that is about 1 mm below the epithelium's outer surface. It is best to remove as little connective tissue as possible. Both a butt joint and a beveled margin can be used to prepare the recipient site's margins. While the donor tissue is being dissected, the recipient site should be prepped and wrapped with surgical gauze soaked with isotonic saline.

Planning in graft preparation

The graft should often be produced a few millimeters longer and wider than what is needed at the recipient site. A knife is used to delineate the graft's size on the palate, and minor bleeding is induced to define the boundaries of the surface. The boundaries of the graft must be arranged so that its thinner sections are high in the palatal vault or in the first molar area to prevent interference with the palatine artery. It is best to extract the thicker parts from the premolar regions [38].

Dissection of donor tissue

To fit the shape of the ridge defect, the graft's base should be V- or U-shaped. Therefore, the several palate incision planes must converge toward a region beneath the donor site's center or toward one of its edges. Using a scalpel to dissect in an anteroposterior manner or from a region high in the palate in a lateral direction towards the teeth is quite simple. Dissecting from the donor site's distal margin in an anterior direction is challenging. The donor tissue must always be kept in pieces of surgical gauze that have been moistened with isotonic saline after it has been removed [39].

Treatment of the donor site

An acrylic stent should be made prior to surgery because it is challenging to anchor and maintain a periodontal dressing at the donor site in the palatal vault. It is necessary to closely examine the donor site for indications of arterial hemorrhage. A circumferential suture must be positioned around the vessel distal to the bleeding spot if any minor vessel bleeding is noticed. The void at the donor site should then be filled with an appropriate hemostatic substance, and sutures should be used to close the wound's edges. After that, the stent is positioned [40].

Try-in and stabilization of graft

For a try-in, the graft is moved to the recipient site using tissue forceps. To match the connective tissue surface of the prepared ridge, the graft is trimmed to the appropriate form and adjusted. Before suturing, a number of parallel incisions may be made deep into the exposed lamina propria at the recipient site to cut major blood arteries. Along the edges of the graft, a number of interrupted sutures are positioned [41].

Wound healing in the recipient site

During the first week following pouch and onlay augmentation surgeries, significant postoperative edema frequently happens. A white layer will develop on the graft's surface when the epithelium sloughs off. During the first week following surgery, patients should rinse with an antimicrobial mouthwash two to four times a day. They should also avoid using mechanical cleaning methods in the area until a new layer of epithelium has formed over the graft, which would not happen until the graft has regained a functional capillary circulation (4-7 days after the surgery). As the epithelium thickens through stratification, the transplanted tissue will take on its natural hue. After three months, the tissue form is often stable, though more shrinkage could happen over the course of many months. Therefore, it is best to wait six months before starting any final restorative therapies [42]. The donor site will progressively fill with granulation tissue. Three weeks following the excision of a 4-5-mm-thick graft, the first healing process is often over. To preserve the healing wound, patients should wear the surgical stent for roughly two weeks. After roughly three months, the palate reverts to its preoperative shape.

Combined onlay-interpositional graft procedures

Because the ridge must be supplemented in both vertical and horizontal dimensions, Class III ridge defects present significant challenges for the physician. In this case, the combined onlay-interpositional graft technique may be applied with success. The mixed graft procedure can be beneficial in many ways. The following benefits could be provided by the mixed graft procedure. The onlay portion of the interpositional graft is revascularized with the help of the submerged connective tissue segment, increasing the total take percentage of the graft. There is a smaller open wound in the palate donor site following surgery.

The technique causes less discomfort for the patient and quicker healing at the palate donor site. It has more flexibility or control over the extent of apicocoronal and buccolingual augmentation with a single treatment. The mucogingival junction is neither displaced coronally nor is the vestibular depth reduced. Therefore, subsequent corrective measures are not required.

Refinement of pontic contours and gingivoplasty soft tissue sculpting procedures

When repairing flaws in a partially edentulous ridge, it is preferable to slightly overcorrect the ridge where the deformity occurs. In addition to compensating for wound contraction, this will provide the ridge with the bulk of tissue it needs to take on its ultimate shape. To smooth out incision lines and achieve the ideal fit and contour of the pontic teeth to the crest of the ridge, gingivoplasty techniques employ rotational coarse diamond stones in an ultra-speed handpiece with ample water spray. The emerging profile and cervical contour of the pontic are adjusted to resemble the contralateral teeth. The tissue-contacting surfaces of the pontic teeth are instantly polished and rebased with autopolymerizing resin. Although the final tissue sculpting process and the reshaping of the temporary prosthesis are minimal, they are very helpful in defining the papillae's shape and giving the appearance that there is a cuff of free gingiva at the pontic/ridge contact.

Selection of the soft tissue augmentation method

If the localized ridge defect is accompanied by other mucogingival-esthetic issues, such as narrow keratinized gingiva, high lip or cheek frenum, gingival tattoo, or unesthetic gingival contour, all of the issues can be resolved with a single surgical procedure using the free full-thickness onlay graft. However, there is a chance that this method will only result in a partial volumetric success. A second intervention involving a subepithelial connective tissue graft would thereafter be necessary. The onlay transplant is recommended in cases when there are only volumetric defects and no other mucogingival-esthetic issues since it offers a more definite prognosis in terms of the increased volume. To benefit from its advantageous single operating site, the roll flap procedure can be applied to modest defects (less than 3 mm). Because more tissue may be extracted from the roof of the palate using the subepithelial transplant than with the roll flap technique, it should be utilized for moderate to severe (at least 4 mm) lesions and even less severe ones for whom the roll flap approach is inappropriate [3,5]. The indications and contraindications of soft tissue augmentation are listed in Table 7.

**Table 7:** Indications and contraindications of soft tissue augmentation

Indications	Contraindications
When a patient wants a fixed restoration for maxillary Kennedy Class IV anterior tooth loss and there is a mild to severe ridge defect, a soft tissue transplant can be used to widen the prescription for an esthetic fixed partial denture rather than a full denture	When the top lip line during laughter does not expose the ridge defect in a visible area. However, it is likely that only a small percentage of people have a deep smile line that conceals no part of the mucogingival complex
To make a pontic or single-tooth implant more esthetically pleasing	When the patient has no interest in esthetics or lacks dental motivation
For the plastic surgical repair of ridge deficiencies that result from peri-implant surgery or the loss of a single-tooth implant	When the neighboring teeth exhibit deep pockets and/or bleed when probed
To get rid of unsightly gingival texture or gingival tattooing	A delicate surgical approach is essential to the procedure's effectiveness when the operator lacks expertise doing periodontal surgery
Rarely, a soft tissue implant can be used to remove an undercut area in the direction of insertion when a partial denture is planned, avoiding the more invasive ostectomy	If there is a severe deficiency affecting three or four teeth, it should be fixed using a bone augmentation technique

Clinical relevance of soft tissue augmentation

The following conclusions on soft tissue augmentation for implants were offered by a recent systematic study that thoroughly gathered data from numerous randomized controlled trials (RCTs) and other systematic reviews across time [43,44]. Compared to no augmentation, soft tissue augmentation in the esthetic zone causes the mid-buccal mucosa to thicken and causes less recession of the mid-buccal mucosa in delayed implant placement. When compared to the lack of soft tissue augmentation, soft tissue augmentation has a limited impact on changes at the marginal bone level. Augmenting the keratinized mucosa enhanced the gingival index, mucosal recession, and plaque management. The use of connective tissue grafts to increase soft tissue volume improves cosmetic criteria.

When compared to hard tissue augmentation alone, sites treated with both soft and hard tissue augmentation show no statistically significant differences in marginal bone-level alterations. Compared to hard tissue augmentation alone, concurrent soft and hard tissue augmentation produced reduces marginal soft tissue recession. It is unknown if soft tissue augmentation techniques improve the short- and long-term results of implant therapy more than hard tissue regeneration or if they have any other favorable effects. Limited clinical evidence suggests that connective tissue grafts for soft tissue augmentation produce stable peri-implant soft tissues during a medium-term follow-up.

Timing of soft tissue augmentation

Soft tissue augmentation may be advised concurrently with rapid implant placement in cases of high esthetic priority in order to decrease soft tissue recession and thicken the mucosa. To lessen soft tissue recession in situations with a high cosmetic priority, soft tissue augmentation combined with postponed implant placement may be advised. There is no way to provide therapeutic advice regarding the usage of soft tissue replacements. The best time point for soft tissue augmentation cannot be determined clinically. There is insufficient data to determine the ideal time point for soft tissue augmentation at implant locations [45].

Limitations

Although soft tissue augmentation methods yield encouraging short- to medium-term outcomes, there is a dearth of long-term follow-up evidence. More research is needed to determine whether the enhanced tissues surrounding implants will remain stable over time. Furthermore, a patient's age, systemic health issues, genetics, smoking habits, and dental cleanliness all have a significant impact on the outcome of soft tissue augmentation. These factors might make it more difficult to forecast results and make generalizations. Additionally, depending on the particular clinical scenario, soft tissue augmentation techniques might differ significantly (e.g., single tooth implants vs. whole arch restorations). Treatment techniques become confused and inconsistent when there are no defined rules that are particular to a certain indication.

Recommendations

To evaluate the durability of soft tissue grafts around implants, especially their resistance to atrophy or recession over time, more extensive research is required. This will improve our comprehension of how long-lasting certain augmentation techniques are. When designing soft tissue augmentation operations, doctors must also take into account the unique features of each patient, such as bone volume, soft tissue phenotype, and esthetic requirements. A customized strategy can reduce problems and maximize clinical results. Advanced imaging methods (such as optical scanning and 3D cone beam computed tomography) might help with a more precise evaluation of the soft tissue and surrounding structures when used in preoperative planning. This will aid in forecasting the results and problems of surgery. It would be beneficial to conduct more studies on methods that enhance the esthetic results of soft tissue augmentation, such as better color matching, contouring, and texture of the supplemented tissue. This may result in a better concord between the surrounding natural tissues and the implant.

Future directions

One intriguing avenue is the use of regenerative medicine with implant dentistry. The regeneration of soft tissues surrounding implants may be improved by the application of growth factors, stem cells, or bioactive scaffolds, which might provide more consistent and long-lasting results. As technology progresses, there is growing interest in minimally invasive methods to shorten recovery periods and minimize patient suffering, such as the use of bioresorbable membranes or laser-assisted soft tissue augmentation. Clinicians may be able to make better treatment decisions and provide better patient-specific care if they employ artificial intelligence and machine learning algorithms to forecast the results of soft tissue augmentation treatments. The investigation of more recent biomaterials, including biodegradable scaffolds or artificial materials that replicate the behavior of natural tissue, may present viable substitutes for the materials used in grafting, which might enhance the long-term viability and visual integration of soft tissue augmentations.

## Conclusions

Soft tissue augmentation plays a pivotal role in implant dentistry, particularly in cases where achieving optimal esthetics and long-term stability is of paramount importance. The integration of various augmentation techniques, such as pedicle grafts, free connective tissue grafts, and onlay grafts, has significantly improved clinical outcomes by enhancing the volume and contour of peri-implant tissues. The selection of the appropriate technique depends on the severity and extent of ridge defects, with considerations given to both functional and esthetic demands. Studies have demonstrated that soft tissue augmentation contributes to reducing mucosal recession, improving keratinized tissue width, and ensuring long-term peri-implant health. Despite advancements in surgical techniques and biomaterials, challenges remain in determining the optimal timing and method for augmentation, especially in cases requiring combined hard and soft tissue interventions. Future research should focus on refining augmentation protocols, optimizing patient-specific treatment planning, and exploring the long-term effects of soft tissue augmentation on implant survival and esthetic outcomes. With evolving clinical evidence, the role of soft tissue management in implant dentistry continues to expand, underscoring its significance in achieving predictable and successful implant restorations.
